# The impact of S6K1 kinase on neuroblastoma cell proliferation is independent of GLI1 signaling

**DOI:** 10.1186/1471-2407-14-600

**Published:** 2014-08-18

**Authors:** Yumei Diao, Mohammed Ferdous-Ur Rahman, Victoria E Villegas, Malin Wickström, John I Johnsen, Peter G Zaphiropoulos

**Affiliations:** Department of Biosciences and Nutrition, Karolinska Institutet, Huddinge, Sweden; Faculty of Natural Sciences and Mathematics & Doctoral Program in Biomedical Sciences Universidad del Rosario, Bogotá, Colombia; Department of Women’s and Children’s Health, Childhood Cancer Research Unit, Karolinska Institutet, Solna, Sweden

**Keywords:** Hedgehog signaling, Protein phosphorylation, Signaling pathway crosstalk, Cellular proliferation, Cell growth, Oncogenic signaling, mTOR/S6K1 signaling, Signaling inhibitors

## Abstract

**Background:**

The crosstalk between Hedgehog (HH) signaling and other signal transduction cascades has been extensively studied in different cancers. In neuroblastoma, mTOR/S6K1 signaling is known to have a role in the development of this disease and recent evidence also implicates the HH pathway. Moreover, S6K1 kinase has been shown to phosphorylate GLI1, the effector of HH signaling, promoting GLI1 transcriptional activity and oncogenic function in esophageal adenocarcinoma. In this study, we examined the possible interplay of S6K1 and GLI1 signaling in neuroblastoma.

**Methods:**

siRNA knockdowns were used to suppress S6K1 and GLI1 expression, and the siRNA effects were validated by real-time PCR and Western blotting. Cell proliferation analysis was performed with the EdU incorporation assay. Cytotoxic analysis with increasing concentrations of PI3K/mTOR and GLI inhibitors, individually and in combination, was used to determine drug response.

**Results:**

Although knockdown of either S6K1 or GLI1 reduces the cellular proliferation of neuroblastoma cells, there is little effect of S6K1 on the expression of GLI1 mRNA and protein and on the capacity of GLI1 to activate target genes. No detectable phosphorylation of GLI1 is observed prior or following S6K1 knockdown. GLI1 overexpression can not rescue the reduced proliferation elicited by S6K1 knockdown. Moreover, inhibitors of PI3K/mTOR and GLI signaling reduced neuroblastoma cell growth, but no additional growth inhibitory effects were detected when the two classes of drugs were combined.

**Conclusion:**

Our results demonstrate that the impact of S6K1 kinase on neuroblastoma cells is not mediated through modulation of GLI1 expression/activity.

**Electronic supplementary material:**

The online version of this article (doi:10.1186/1471-2407-14-600) contains supplementary material, which is available to authorized users.

## Background

Neuroblastoma is the most common and deadly tumor of infancy [1, 2]. It accounts for about 10% of childhood cancers and the mortality reaches 12% [1, 3, 4]. Despite a better understanding of the molecular, cellular and genetic events that can lead to neuroblastoma development there is still a need to explore new druggable targets for this disease.

The Hedgehog (HH) signaling pathway has critical roles in embryonic development and tumorigenesis [5–8]. Aberrant activation of HH signaling is involved in several types of malignant tumors, including medulloblastoma, rhabdomyosarcoma, basal cell carcinoma, and cancers of the pancreas, colon, stomach, lung and prostate [9–11]. The pathway is initiated by HH ligand [Sonic HH (SHH), Indian HH (IHH), Desert HH (DHH)] [12, 13] binding to Patched (PTCH1, PTCH2), a twelve trans-membrane domain receptor protein. In the absence of ligands, PTCH inhibits the signaling of the seven trans-membrane domain protein, the proto-oncogene Smoothened (SMO). Upon HH binding, the inhibition of PTCH on SMO is relieved and the signal is transduced to the terminal effectors, the GLI (GLI1, GLI2, GLI3) transcription factors [12–16]. GLI1 not only acts as a signaling effector but also represents a pathway target gene [16], amplifying the HH signal. Its expression levels are thus a good marker of pathway activity.

Recent studies indicate that primary neuroblastoma and neuroblastoma cell lines express high levels of proteins involved in HH signaling [17–19]. Additionally, inhibition of this pathway at the level of GLI1 is more potent than SMO blockade in reducing the cellular proliferation of non-MYCN amplified neuroblastoma cell lines [19]. This suggests that GLI1 inhibition of HH signaling is an effective way to target high-risk neuroblastoma without MYCN amplification and should be considered as an option for neuroblastoma treatment.

The mammalian target of rapamycin (mTOR) has emerged as a critical effector in cell signaling pathways commonly deregulated in human cancers. mTOR regulates cell growth by controlling mRNA translation, ribosome biogenesis, autophagy, and metabolism [20]. Specifically, mTOR regulates translation by the phosphorylation of the ribosomal p70S6 kinase 1 (S6K1), which promotes cap-dependent translation through phosphorylation of eukaryotic translation initiation factor 4E-binding protein 1 (4E-BP1) [21]. Full and sustained S6K1 activation requires phosphorylation at amino acid residues T229, located within the catalytic activation loop, and T389, located at the hydrophobic motif [22]. Furthermore, the phosphorylated and activated form of S6K1 (T389) is decreased after treatment with the mTOR inhibitors rapamycin or CCI-779 in neuroblastoma cells [23]. Additionally, the PI3K/mTOR inhibitor PI103 induced time- and concentration-dependent inhibition of cell growth in both MYCN and non-MYCN amplified neuroblastoma cell lines [24].

Recently, the mTOR/S6K1 pathway was shown to mediate the development of esophageal adenocarcinoma (EAC) through GLI1 signaling [25]. Activation of the mTOR/S6K1 pathway via S6K1 phosphorylation was demonstrated to phosphorylate GLI1, promoting GLI1 transcriptional activity and oncogenic function.

In this context, we explored if a crosstalk between mTOR/S6K1 and HH signaling is relevant in neuroblastoma. Our data provide little support for a role of GLI1 signaling as a mediator of the S6K1 proliferative effects in neuroblastoma cells. S6K1 knockdown has minimal effects on GLI1 signaling, GLI1 overexpression can not rescue the reduced proliferation elicited by S6K1 knockdown, and combinations of mTOR/S6K1 and GLI inhibitors do not reveal additive or synergistic effects. Thus, we conclude that S6K1 and GLI1 signaling exert proliferative effects on neuroblastoma cells through independent mechanisms.

## Methods

### siRNAs and plasmids

siRNAs against S6K1 (RPS6KB1) (NCBI Reference Sequence: NM_003161.3) were designed and ordered from Dharmacon (SiGenome SMART pools, Thermo Scientific). GLI1 siRNAs and control siRNAs were purchased from Sigma-Aldrich.

S6K1 overexpression plasmids, wild type plasmid S6K1WT, constitutively activated plasmid S6K1T389E and function-loss plasmid S6K1T389A were kind gifts of Mien-Chie Hung (University of Texas, MD Anderson Cancer Center, Houston, TX). The GLI1 expression construct (Flag-tagged) has been described previously [15].

### Cell culture

Neuroblastoma cell lines SK-N-AS (non-MYCN-amplified, high GLI1 expression) and SK-N-BE(2) (MYCN-amplified, low GLI1-expression) [19, 23, 24], obtained from ATCC (Manassas, VA), were cultured in RPMI-1640 with 10% fetal calf serum and 100 IU/ml penicillin/streptomycin and maintained in a 5% CO_2_ humidified incubator. RPMI-1640, penicillin/streptomycin, and trypsin were purchased from Invitrogen. Recombinant tumor necrosis factor alpha (hTNF-α) was obtained from Roche Applied Sciences.

### Transfection of siRNAs and expression constructs

Cells were plated in 6-well plates (5 × 10^5^ cells per well) or 10 cm^2^ dishes (3 × 10^6^ cells per dish), and transfections were performed with Lipofectamine 2000 (Invitrogen) according to the manufacturer’s protocol (5 μl Lipofectamine reagent per well for 6-well plate, and 10 μl for 10 cm^2^ dish). After each treatment, cells were incubated at 37°C for 6 hours followed by a change to fresh culture medium. Transfection efficiencies were confirmed by siGLO (Green Transfection Indicator, Dharmacon). To evaluate the effect of TNF-α, cells, after a 48-hour transfection and overnight starvation, were treated with TNF-α (5 ng/ml) for 6 hours. Cells were harvested 48 or 72 hours after transfection for cell proliferation assay, mRNA and protein analysis.

### Cell proliferation

5 × 10^5^ cells per well were seeded in 6-well plates, treated with siRNAs for 48 hours, followed by a 4 hour 10 μM EdU (5-ethynyl-2′deoxyuridine) incubation. EdU were detected by fluorescent-azide coupling reaction (Click-iT, Invitrogen). For each treatment, 10 000 cells were analyzed on a FACS calibur machine (BD Biosciences, Stockholm, Sweden). Cell cycle distribution was calculated using the CellQuest software (BD Bioscience). All proliferation experiments were done at least in triplicate and representative experiments are shown.

### Cell survival analysis

For cytotoxic evaluation, we used the fluorometric microculture cytotoxicity assay (FMCA), described in detail previously [26]. Cells were seeded into drug-prepared 96- or 384-well microplates (SK-N-AS: 0.055×10^6^ cells/ml, SK-N-BE(2): 0.028×10^6^ cells/ml) and incubated for 72 hours. The cells were washed, fluorescein diacetate was added and after 40 minutes incubation, fluorescence was measured. Cell survival is presented as survival index (SI, %). The studies were designed as suggested in the CalcuSyn software manual, using a fixed molar ratio between the drugs (GANT61:AR-12 20:1; GANT61:CCI-779 2:1 and GANT61:NVP-BEZ235 100:1), intended to be equipotent. The IC_50_ values (inhibitory concentration 50%) were determined from log concentration-effect curves in GraphPad Prism (GraphPad Software) using non-linear regression analysis. Comparison between two groups was made with *t*-test.

### RNA preparation, cDNA synthesis and real-time PCR

Total RNA was isolated with the RNeasy mini kit (Qiagen, Hamburg, Germany) according to the manufacturer’s protocol. cDNA synthesis was performed with random N6 primers (New England Biolabs) and Superscript III (Invitrogen). Real-time PCR was carried out with Power SYBR Green (Applied Biosystems, Foster City, CA) on a 7500 fast real-time PCR system (Applied Biosystems) with primers designed to detect S6K1, GLI1, GLI2, GLI3, SMO and PTCH2 (Table 1). All amplifications were run at least in triplicate and the fold change was normalized to the expression of TATA binding protein (TBP). The relative expression was determined by the ∆Ct method. All RNA expression experiments were done at least in triplicate and representative experiments are shown.

**Table 1:** **Primers for qPCR analysis**

Primer name	Sequence
S6K1	5′ ACATGCTGACTGGAGCACCCCCAT
	5′ GGCTTCTTGTGTGAGGTAGGGAGGCA
GLI1	5′ AGCTACATCAACTCCGGCCAATAGGG
	5′ TGCTGCGGCGTTCAAGAGAGACTG
GLI2	5′ GACATGCGACACCAGGAAGGAAGGT
	5′ GCCGGATCAAGGAGATGTCAGAGATG
GLI3	5′ TGGACCCCAGGAATGGTTACATGGAG
	5′ TGCAATGGAGGAATCGGAGATGGAT
SMO	5′ TTTCTGTCACCCCTGTGGCAACTCC
	5′ CGGGCACACCTCCTTCTTCCTCTTC
PTCH2	5′ TCTTTCTGGGACTGTTGGCCTTTGG
	5′ CCTCCCCCAGCTTCTCCTTGGTGTA
TBP	5′ GCCAGCTTCGGAGAGTTCTGGGATT
	5′ CGGGCACGAAGTGCAATGGTCTTTA

### Western blot

For Western blotting, cells were lysed with RIPA buffer (150 mM NaCl, 50 mM Tris base pH 8.0, 1 mM EDTA, 0.5% sodium deoxycholate, 1% NP-40, 0.1% sodium dodecyl sulfate, 1 mM DTT, 1 mM PMSF, and 1 mM Na_3_VO_4_) supplemented with Complete Protease Inhibitor Tablets (Roche) and phosphatase inhibitor (Sigma). Proteins were separated on a 7.5% sodium dodecyl sulfate polyacrylamide gel electrophoresis (PAGE) followed by transfer (220 mA for 1 hour) to an Immobilon-P membrane (Millipore). The membrane was incubated at 4°C overnight in 5% skim milk in TBST (Tris Buffered Saline with Tween 20) with anti-rabbit GLI1 Ab (#2553, Cell Signaling) or anti-rabbit S6K1 Ab (sc-230, Santa Cruz Biotechnology) followed by incubation with goat anti-rabbit secondary antibodies for 1 hour in 5% skim milk in TBST and visualized using chemiluminescent substrate (Thermo Scientific). The Western blot experiments were done at least in triplicate and representative experiments are shown.

### Immunoprecipitation (IP)

For immunoprecipitation, cell lysates were generated with lysis buffer (25 mM Tris, pH 7.4, 150 mM NaCl, 1 mM EDTA, 5% glycerol, 1% NP-40 and Protease/phosphatase inhibitor cocktail), and proteins immunoprecipitated using anti-rabbit GLI1 Ab or healthy rabbit serum and Protein A-Agarose according to the manufacturer’s protocol (Santa Cruz Biotechnology). The protein/antibody/Protein A-Agarose complex was washed with PBS containing 0.05% Tween 20. Cell lysates and immunoprecipitated proteins on the transferred membrane were incubated with anti-mouse GLI1 Ab (#2643, Cell Signaling) for GLI1 or anti-mouse phosphoserine/threonine Ab (612548, BD Transduction Laboratories) for phosphorylated GLI1, followed by incubation with goat anti-mouse Ab. The immunoprecipitation experiments were done at least in triplicate and representative experiments are shown.

## Results

### S6K1 knockdown reduces cell proliferation

**Figure 1:**
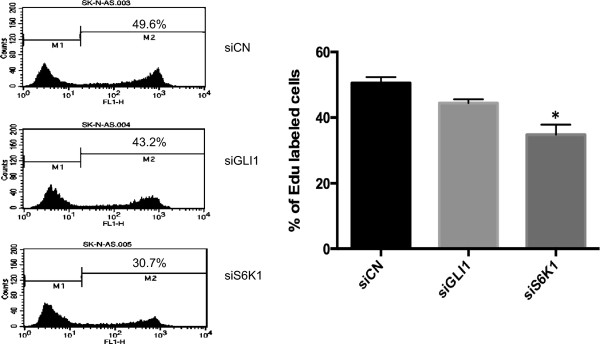
**S6K1 and GLI1 knockdown reduces SK-N-AS cellular proliferation.** SK-N-AS cells, cultured for 48 hours following transfection with control (siCN), GLI1 (siGLI1) or S6K1 (siS6K1) siRNAs, were subjected to the EdU incorporation assay for 4 hours. The percentage of cells labeled with Alexa Fluor 488 azide was detected by flow cytometry. The data were analyzed with the one-way ANOVA test followed by Tukey’s multiple comparison using the GraphPad Prism software. Each bar represents the mean ± SEM of three independent experiments. *, Statistical significant, P < 0.05 compared to control. One representative experiment is shown in the histograph. Note that treatment with the S6K1 siRNAs is more effective than the GLI1 siRNAs in reducing cellular proliferation.

**Figure 2:**
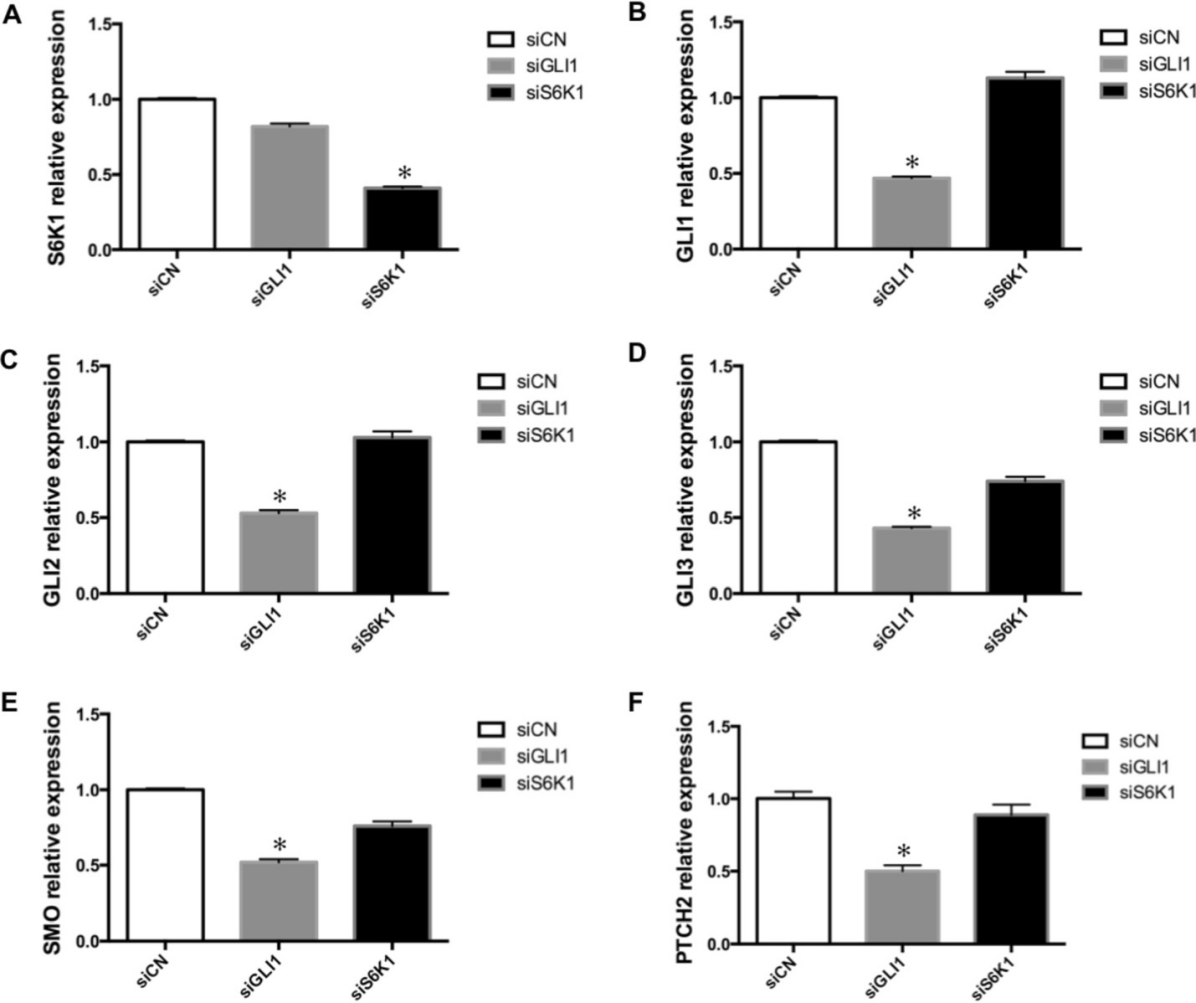
**GLI1 but not S6K1 knockdown reduces GLI1, GLI2, GLI3, SMO and PTCH2 expression.** The expression of S6K1 **(A)**, GLI1 **(B)**, GLI2 **(C)**, GLI3 **(D)**, the signaling molecule SMO **(E)** and the typical GLI1 target gene PTCH2 **(F)** in SK-N-AS cells, following siRNA knockdown of GLI1 and S6K1, was determined by real-time PCR. Data are represented as relative expression (2^-∆∆Ct^ values), calculated by subtracting the Ct value of the housekeeping gene *TBP* from the Ct value of the interrogated transcripts (∆Ct), and normalized to the ∆Ct value obtained with siCN. Error bars indicate the standard deviation. *, Statistical significant, P < 0.02 compared to control, calculated by the Student’s *t*-test.

**Figure 3:**
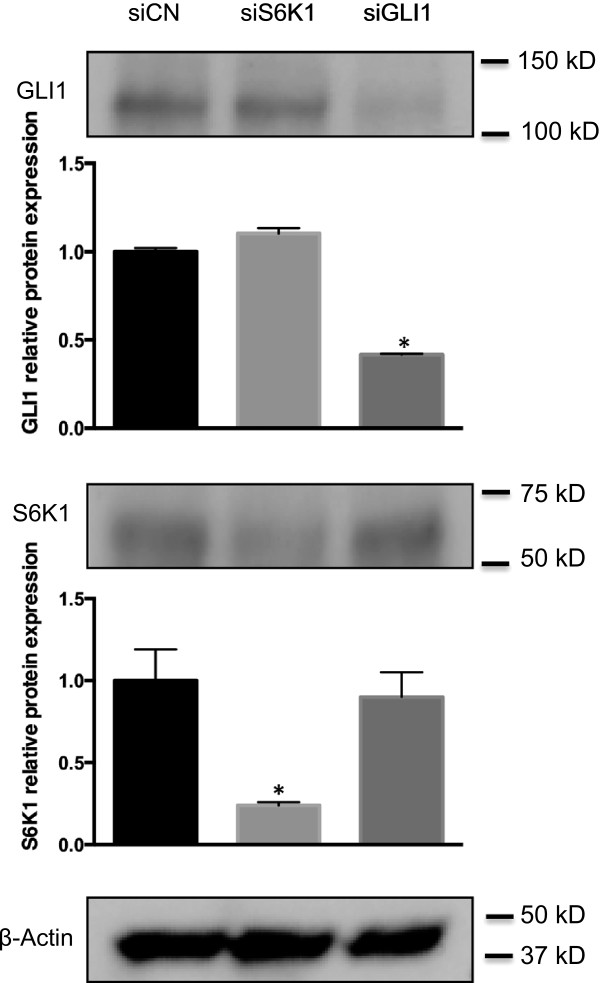
**GLI1 protein levels are unchanged following S6K1 knockdown.** Western blot analysis of GLI1 and S6K1 protein expression in SK-N-AS cells, following siRNA-mediated knockdown of GLI1 and S6K1. Note the reduction of the GLI1 and S6K1 protein bands by the siRNAs targeting GLI1 (siGLI1) and S6K1 (siS6), respectively. siCN indicates the control siRNA treatment and β-Actin was used as the endogenous protein control. Quantitation of protein expression, using the ImageJ software, is shown in the bar graphs. Each bar represents the mean ± SEM of triplicate values from a representative experiment. *, Statistical significant, P < 0.01 compared to control, calculated by the Student’s *t*-test using the GraphPad Prism software.

To investigate the role of S6K1 and GLI1 in neuroblastoma cellular proliferation, we first transfected SK-N-AS cells with siRNAs targeting S6K1 or GLI1. This cell line was chosen to initiate the analysis because of our previous finding that its growth is most sensitive to GLI1 inhibition [19]. 48 hours after transfection cell proliferation was analyzed using FACS. Introduction of S6K1 siRNAs into SK-N-AS cells reduced cellular proliferation compared to the corresponding siRNA control (Figure 1). Moreover, GLI1 siRNAs treatment also decreased proliferation but not to the same extent as the S6K1 knockdown. Considering that the knockdown of GLI1 and S6K1 kinase, determined by real-time PCR analysis (Figure 2A and B) and Western blotting (Figure 3) is comparable, we conclude that S6K1 silencing has stronger effects on SK-N-AS cellular proliferation than GLI1 silencing.

### GLI1, GLI2, GLI3, SMO and PTCH2 expression is insensitive to S6K1 knockdown

To explore the biological mechanisms of S6K1 on SK-N-AS cell proliferation and address the possible involvement of HH signaling, we measured the RNA expression of several key components of this pathway (GLI1, GLI2, GLI3, SMO and PTCH2) following siRNA-mediated knockdown of S6K1. Although the results clearly showed that the S6K1 and GLI1 siRNAs reduced the expression of S6K1 and GLI1, respectively, the effects on the HH signaling components were distinctly different. GLI1 knockdown decreased the expression of the signaling molecule SMO, the effectors GLI2 and GLI3, and PTCH2, which is known to act as a target gene of the pathway [27], while this was not the case with S6K1 knockdown (Figure 2). Similarly, PTCH1, another target gene, is reduced by GLI1 but not S6K1 knockdown (data not shown). Importantly, GLI1 expression was unaffected by knocking down S6K1. Thus, the mechanism of S6K1 on SK-N-AS cell proliferation is, apparently, not related to the expression the HH signaling components analyzed.

Moreover, the use of the SK-N-BE(2) neuroblastoma cell line (Methods), which has low GLI1 expression compared to SK-N-AS cells, also demonstrated that S6K1 knockdown has no effect on GLI1 mRNA levels. Finally, treatment of either SK-N-AS or SK-N-BE(2) cells with TNF-α, a cytokine that can induce S6K1 activity, failed to show any S6K1 dependence on GLI1 expression (Additional file 1: Figure S1).

### S6K1 knockdown does not alter GLI1 protein levels and has no detectable impact on GLI1 phosphorylation

In esophageal adenocarcinoma, S6K1 was demonstrated to have the capacity to phosphorylate GLI1 increasing its transcriptional activity [25]. We, therefore, tested whether GLI1 may be subjected to S6K1-dependent phosphorylation in SK-N-AS cells. Initially, the protein levels of GLI1 were determined by Western blotting, revealing comparable expression prior and following S6K1 knockdown (Figure 3). Subsequently, immunoprecipitation analysis confirmed that the protein expression of GLI1 is not altered by knocking down S6K1. Moreover, no GLI1 phosphorylation was observed, irrespective of the status of the S6K1 (Additional file 1: Figure S2). Thus, in SK-N-AS cells, S6K1-dependent phosphorylation of GLI1 is not taking place at detectable levels.

### GLI1 overexpression can not rescue the reduced cell proliferation elicited by S6K1 knockdown

**Figure 4:**
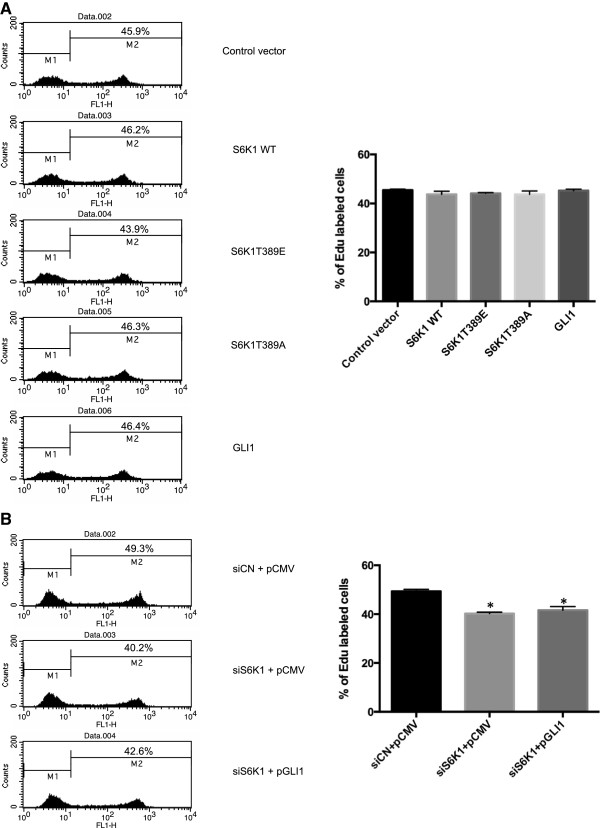
**SK-N-AS cellular proliferation is insensitive to S6K1 or GLI1 overexpression. (A)** SK-N-AS cells, cultured for 48 hours following transfection with control pCMV5 vector, and expression constructs for wild type S6K1 (S6K1 WT) constitutively activated S6K1 (S6K1T389E), function-loss S6K1 (S6K1T389A) and GLI1, were subjected to the EdU incorporation assay for 4 hours. **(B)** SK-N-AS cells, cultured for 48 hours following transfection with control siRNAs and pCMV5 vector (siCN + pCMV), S6K1 siRNAs and pCMV5 vector (siS6K1 + pCMV) and S6K1 siRNAs and GLI1 expression construct (siS6K1 + pGLI1), were subjected to the EdU incorporation assay for 4 hours. The data were analyzed with the one-way ANOVA test followed by Tukey’s multiple comparison using the GraphPad Prism software. Each bar represents the mean ± SEM of three independent experiments *, Statistical significant, P < 0.01 compared to control. One representative experiment is shown in the histographs. For both **(A)** and **(B)** the percentage of cells labeled with Alexa Fluor 488 azide was detected by flow cytometry. Note that overexpression of GLI1 can not rescue the reduced proliferation elicited by knocking down S6K1.

Since knockdown of S6K1 causes a reduction in SK-N-AS cellular proliferation, we asked whether overexpression of S6K1 might affect the proliferation of these cells. However, ectopic expression of S6K1, the constitutively activated mutant S6K1T389E or the function-loss mutant S6K1T389A in SK-N-AS cells could not confer changes in cellular proliferation (Figure 4A), even though protein expression was readily detected by Western blotting (Additional file 1: Figure S3). This is in contrast to the observations in EAC, where overexpression of S6K1 increased cell proliferation [25]. Similarly, GLI1 overexpression did not augment proliferation (Figure 4A), again in contrast to the EAC cells [25]. Consequently, our data suggest that the proliferative effects of endogenous S6K1 and GLI1 have reached saturation in the SK-N-AS cell line. Importantly, GLI1 overexpression could not rescue the reduction of cell proliferation elicited by knocking down S6K1 (Figure 4B). Thus, we conclude that the impact of S6K1 on the proliferation of the neuroblastoma SK-N-AS cells is not mediated through GLI1 signaling.

### Combining GLI and PI3K/mTOR inhibitors does not augment the growth reduction of neuroblastoma cells

**Figure 5:**
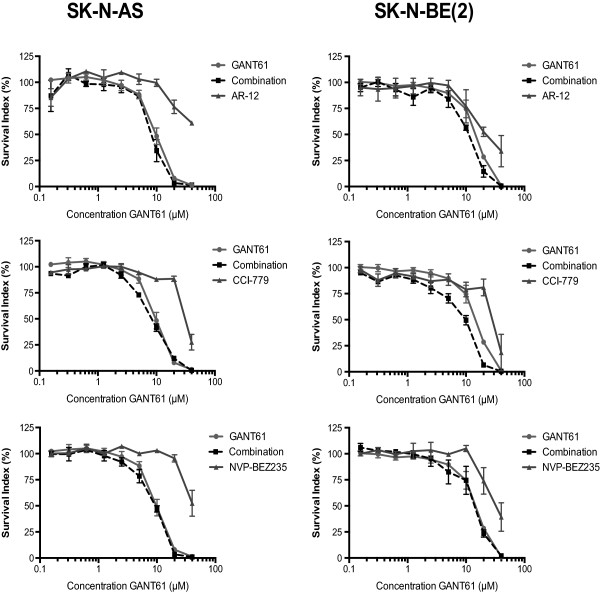
**Combination of small molecule inhibitors of GLI and PI3K/mTOR do not cooperate in inducing the suppression of neuroblastoma cell growth.** Dose–response curves for GANT61 cytotoxicity, in combination with the PI3K/mTOR inhibitors AR-12, CCI-779 and NVP-BEZ 235, in SK-N-AS and SK-N-BE(2) cells treated for 72 h. The PI3K/mTOR inhibitors were tested in the following concentration spans: AR-12: 2 μM - 0.0078 μM, CCI-779: 20 μM – 0.078 μM and NVP-BEZ235: 0.4 μM - 0.0016 μM. A fixed ratio of GANT61 to the PI3K/mTOR inhibitors was used in the combination experiments (GANT61:AR-12 20:1; GANT61:CCI-779 2:1 and GANT61:NVP-BEZ235 100:1). Note that no additive or synergistic effects are seen in the combinatorial treatments except for the GANT61/CCI-779 combination in SK-N-BE(2) cells. This may relate to high concentration of CC1-779 used compared to the other PI3K/mTOR inhibitors, which could elicit non-specific effects in this cellular context.

To further examine the lack of observable interactions between GLI1 and S6K1 signaling, the cytotoxicity of the GLI inhibitor GANT61 [28] and the PI3K/mTOR inhibitors, AR-12 (OSU03012), CCI-779 and NVP-BEZ235 was evaluated using FMCA not only in SK-N-AS but also in SK-N-BE(2) cells, previously shown to be the least dependent on GLI1 signaling [19] (Figure 5). No differences between the log IC_50_ of GANT61 and the log IC_50_ produced by the combination (*t*-test, p > 0.05), except for the combination of GANT61 and CCI-779 in SK-N-BE(2) cells (*t*-test, p = 0.032), was observed (Additional file 1: Table S1).

## Discussion

Deregulation of the HH signaling pathway has long been known to be associated with various human cancers. Recently, neuroblastoma was added to this list based on a series of observations. GLI2, GLI3 and especially GLI1 knockdown reduced neuroblastoma cell growth compared with siRNA control [19]. Moreover, GANT61, a GLI inhibitor, reduced the *in vivo* growth of high-risk neuroblastoma lacking MYCN amplification [19]. These findings extend earlier reports, which indicated that inhibition of HH signaling by cyclopamine induced apoptosis, blocked proliferation and abrogated the tumorigenicity of neuroblastoma cells [18].

The HH signaling pathway is known to interact with other signal transduction cascades during cancer development, exemplified by the TGFβ – HH crosstalk in pancreatic adenocarcinoma [10]. Recently, a connection between the mTOR/S6K1 and the HH pathway has been reported in EAC, through an S6K1-mediated GLI1 phosphorylation at Ser84, which increases its transcriptional/oncogenic activity [25]. It should be noted that the S6K1 impact on GLI1 was observed following TNF-α treatment, which activates S6K1. Without administration of this cytokine there is little detection of active (phosphorylated) S6K1 and phosphorylated GLI1. Furthermore, knocking down S6K1 in HeLa cells had little effect on GLI activity, unless AKT or ERK signaling was activated [25]. In this study, we found that S6K1 knockdown is more effective than GLI1 knockdown in reducing the cellular proliferation of the non-MYCN amplified SK-N-AS cell line. Additionally, knocking down S6K1 did not affect GLI1 expression, irrespective of the treatment of the cells with TNF-α. When the MYCN amplified and lowly GLI1 expressing SK-N-BE(2) neuroblastoma cell line was used, S6K1 knockdown did not change GLI1 expression in the absence of TNF-α. TNF-α treatment increased GLI1 mRNA levels but this upregulation was insensitive to S6K1 knockdown, arguing for the lack of involvement of this kinase. Moreover, we could not detect changes in the phosphorylation status of GLI1 by S6K1 knockdown in SK-N-AS cells. The most likely reason for this is that the endogenous level of phosphorylated GLI1, if any, is beyond the detection limit of the assay used. Another possibility could be that the endogenous level of active S6K1 may be too low to phosphorylate GLI1. However, this is not supported by the fact that overexpression of S6K1 does not elicit proliferation changes, while S6K1 knockdown does, arguing that the endogenous S6K1 levels are sufficient for biological effects. In fact, active (phosphorylated) S6K1 is readily detectable in the SK-N-AS cell line [23]. Thus, our data suggest that GLI1 is not a target of S6K1 and the impact of S6K1 on cellular proliferation is independent of GLI1. This is further supported by the inability of GLI1 overexpression to rescue the reduced proliferation elicited by S6K1 knockdown. Additionally, the combination of small molecule inhibitors of GLI and PI3K/mTOR signaling revealed no additive or synergistic effects on the suppression of neuroblastoma cell growth.

It should be also noted that a recent kinome-wide siRNA screen in a non-small cell lung cancer cell line revealed that S6K1 silencing does not alter the expression of GLI1 protein and GLI1 regulated genes [29], in line with our observations in neuroblastoma. Further analysis examining possible interactions between S6K1 and GLI1 in other cell types will provide additional clarity on these issues.

## Conclusion

Our experimental data demonstrate that in the context of the neuroblastoma cells analyzed S6K1 kinase is not activating Hedgehog signaling through GLI1 phosphorylation. These findings suggest that the effects of S6K1 and GLI1 signaling on neuroblastoma cell proliferation are mediated through independent mechanisms.

## Electronic supplementary material

Additional file 1: Figure S1: GLI1 expression is not S6K1 dependent in control or TNF-α treated SK-N-AS and SK-N-BE(2) cells. The expression of S6K1 **(A)** and GLI1 **(B)** in SK-N-AS and SK-N-BE(2) cells transiently transfected with siCN or siS6K1 followed by treatment with or without TNF-α (5 ng/ml) was determined by real-time PCR as in Figure 2. Error bars indicate the standard deviation. *, Statistical significant, P < 0.05 compared to control, calculated by the Student’s *t*-test. Note, that in SK-N-AS cells TNF-α treatment does not effectively modulate GLI1 expression. In SK-N-BE(2) cells it does, but this GLI1 upregulation is not dependent on S6K1. **Figure S2.** S6K1 knockdown does not change the levels of immunoprecipitated GLI1. SK-N-AS cells were cultured for 48 hours following transfection with control (CN) or S6K1 (S6) siRNAs and cell lysates were subjected to immunoprecipitation with rabbit GLI1 antibodies. Western analysis of lysates and immunoprecipitates was performed with mouse GLI1 antibodies (upper panels) and mouse phosphoserine/threonine antibodies (lower panels). Note the comparable GLI1 levels before and after S6K1 knockdown and the absence of a signal for phosphorylated GLI1. **Figure S3.** Expression constructs of S6K1 produce proteins in SK-N-AS cells. SK-N-AS cells were cultured for 48 hours, following transfection with control pCMV5 vector (pCMV), and expression constructs for wild type S6K1 (S6K1 WT), constitutively activated S6K1 (S6K1T389E) and function-loss S6K1 (S6K1T389A). Western blot analysis of cell lysates was done with a rabbit S6K1 antibody. Note the co-migration of the endogenous and exogenous S6K1 protein bands. Quantitation of protein expression, using the ImageJ software, is shown in the bar graph. **Table S1.** Log IC50 values for GANT61 and combination of GANT61 and PI3K/mTOR inhibitors on neuroblastoma cell lines. (PDF 4 MB)
