# Supplementary material for: The impact of S6K1 kinase on neuroblastoma cell proliferation is independent of GLI1 signaling
Source: BMC Cancer. 2014 Aug 18;14:600. doi: 10.1186/1471-2407-14-600 (PMC4152578; doi:10.1186/1471-2407-14-600)
Supplement: Supplementary file 1 — Additional file 1: Figure S1: GLI1 expression is not S6K1 dependent in control or TNF-α treated SK-N-AS and SK-N-BE(2) cells. The expression of S6K1 (A) and GLI1 (B) in SK-N-AS and SK-N-BE(2) cells transiently transfected with siCN or siS6K1 followed by treatment with or without TNF-α (5 ng/ml) was determined by real-time PCR as in Figure 2. Error bars indicate the standard deviation. *, Statistical significant, P < 0.05 compared to control, calculated by the Student’s t-test. Note, that in SK-N-AS cells TNF-α treatment does not effectively modulate GLI1 expression. In SK-N-BE(2) cells it does, but this GLI1 upregulation is not dependent on S6K1. Figure S2. S6K1 knockdown does not change the levels of immunoprecipitated GLI1. SK-N-AS cells were cultured for 48 hours following transfection with control (CN) or S6K1 (S6) siRNAs and cell lysates were subjected to immunoprecipitation with rabbit GLI1 antibodies. Western analysis of lysates and immunoprecipitates was performed with mouse GLI1 antibodies (upper panels) and mouse phosphoserine/threonine antibodies (lower panels). Note the comparable GLI1 levels before and after S6K1 knockdown and the absence of a signal for phosphorylated GLI1. Figure S3. Expression constructs of S6K1 produce proteins in SK-N-AS cells. SK-N-AS cells were cultured for 48 hours, following transfection with control pCMV5 vector (pCMV), and expression constructs for wild type S6K1 (S6K1 WT), constitutively activated S6K1 (S6K1T389E) and function-loss S6K1 (S6K1T389A). Western blot analysis of cell lysates was done with a rabbit S6K1 antibody. Note the co-migration of the endogenous and exogenous S6K1 protein bands. Quantitation of protein expression, using the ImageJ software, is shown in the bar graph. Table S1. Log IC50 values for GANT61 and combination of GANT61 and PI3K/mTOR inhibitors on neuroblastoma cell lines. (PDF 4 MB) [file 12885_2014_4793_MOESM1_ESM.pdf]

Figure S1

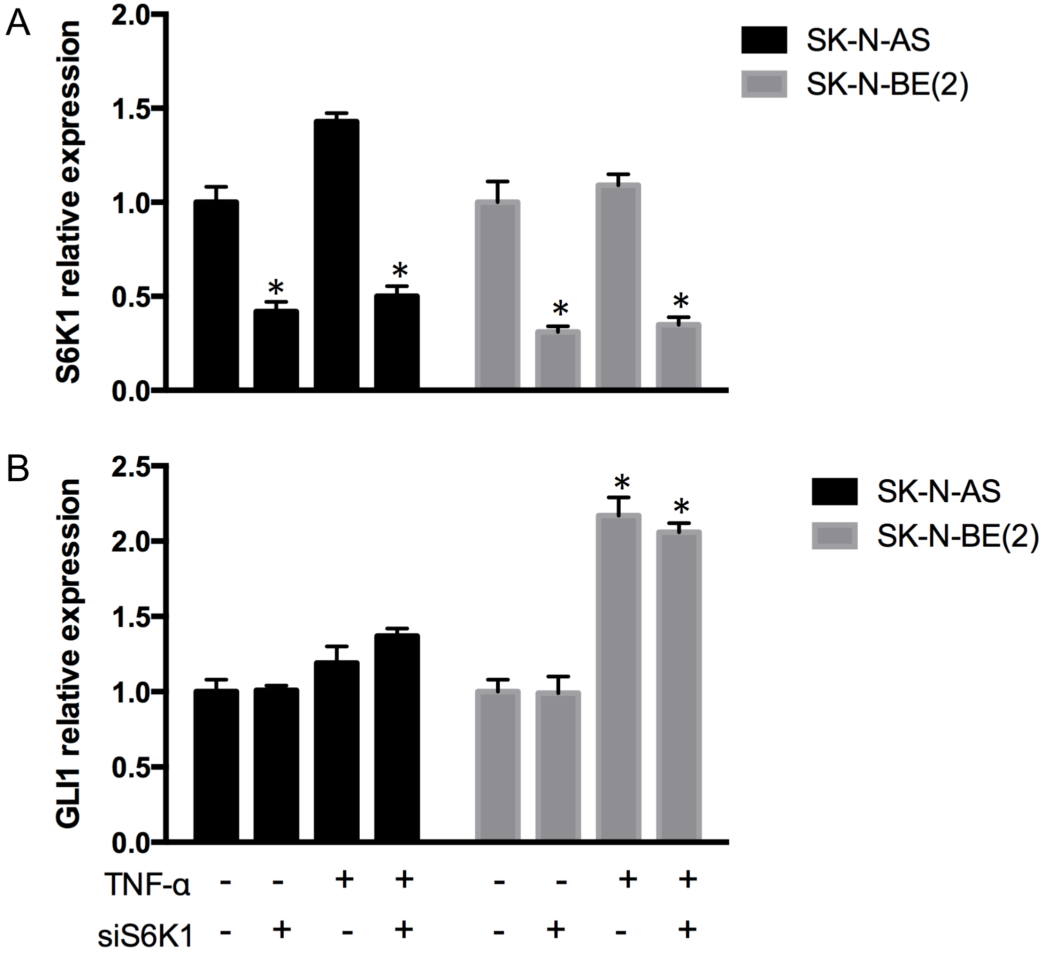
A
S6K1 relative expression
2.0
1.5
1.0
0.5
0.0
SK-N-AS
SK-N-BE(2)
B
GLI1 relative expression
2.5
2.0
1.5
1.0
0.5
0.0
SK-N-AS
SK-N-BE(2)
TNF-α - - + + - - + +
siS6K1 - + - + - + - +

Figure S2

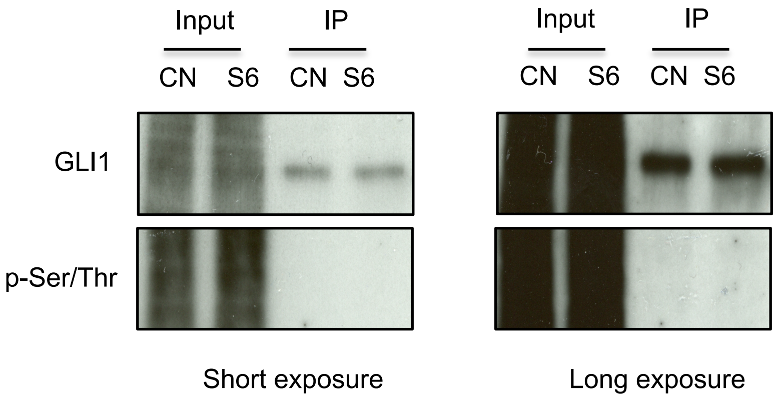
Input
IP
CN
S6
CN
S6
Input
IP
CN
S6
CN
S6
GLI1
p-Ser/Thr
Short exposure
Long exposure

Figure S3

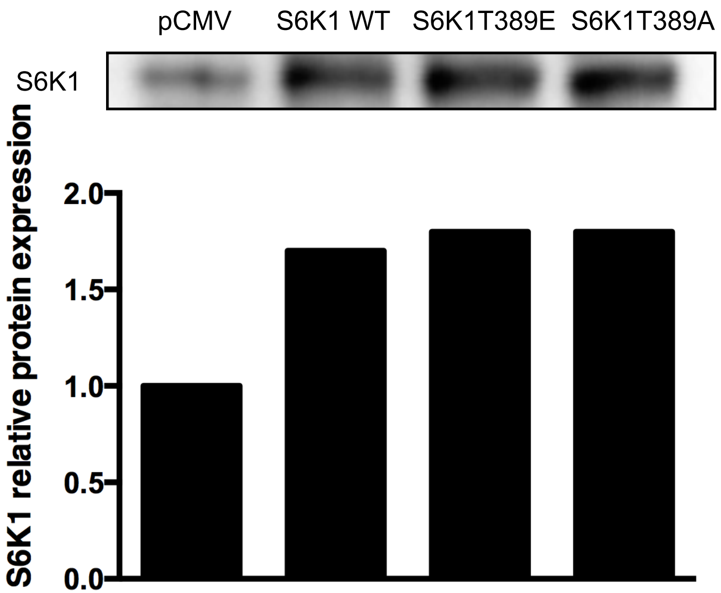

pCMV
S6K1 WT
S6K1T389E
S6K1T389A
S6K1
S6K1 relative protein expression
2.0
1.5
1.0
0.5
0.0

Table S1. Log IC50 values for GANT61 and combination of GANT61 and PI3K/mTOR inhibitors on neuroblastoma cell lines

	SK-N-AS	SK-N-BE(2)
GANT61	0.99	1.15
GANT61 + AR-12	0.92	1.02
GANT61 + CCI-779	0.91	0.89
GANT61 + NVP-BEZ235	0.94	1.13
